# Heart transplantation with generic immunosuppression – a developing country experience

**DOI:** 10.1186/1749-8090-8-S1-O157

**Published:** 2013-09-11

**Authors:** M Villavicencio, V Rossel, R Larrea, J Peralta, J Lim, P Rojo, E Larrain, E Donoso, F Gajardo, M Hurtado

**Affiliations:** 1 Cardiopulmonary Transplant, Instituto Nacional del Torax, Santiago, Chile; 2 Cardiopulmonary Transplant Unit, Clinica Davila, Santiago, Chile

## Background

Heart transplantation is the therapy of choice for advance heart failure. Our group developed two transplant programs at Instituto Nacional del Torax and Clinica Davila. We report our clinical experience based with generic immunosuppression with may have lower costs and may allow more patients to be transplanted.

## Methods

Fifty-three consecutive patients were transplanted between November 2008 and April 2013, representing 51% of all Chilean cases.

## Results

Ischemic or dilated cardiomyopathy were the main indications (23(43%) each), age 48+13 years and 48(91%) were male. Transplant listing Status: IA 14(26%)(VAD or 2 inotropes), IB 14(26%)(1 inotrope) and II 25 (47%) (no inotrope). Mean waiting time 70+83 days. Twelve (24%) were transplanted during VAD support (median support: 36 days). Operative technique: orthotopic bicaval transplant with ischemia time: 175+54 min. Operative mortality: 3 (6%), all due to right ventricular failure. Re-exploration for bleeding 2 (4%), stroke 3(6%), mediastinitis 0(0%), pneumonia 4(8%), and transient dialysis 6 (11%). Mean follow-up was 21+14 months. Three-year survival was 86+6%. One patient died of Pneumocystis Jirovecii pneumonia and the other died suddenly (non-compliance). Freedom from rejection requiring specific therapy was 80+7% at 3 years of follow-up. Four hundred eighty four endomyocardial biopsies were done: 11(2.3%) had 2R rejection. All survivors are in NYHA (New York Heart Association) functional class I and all but one has normal biventricular function.

## Conclusion

Mid-term results are similar to those reported by the registry of the International Society for Heart and Lung Transplantation. Rejection rates are low in spite of generic immunosuppression.

